# Perceptions of Heat Stress, Heat Strain and Mitigation Practices in Wildfire Suppression across Southern Europe and Latin America

**DOI:** 10.3390/ijerph191912288

**Published:** 2022-09-27

**Authors:** Belén Carballo-Leyenda, José Gerardo Villa-Vicente, Giuseppe M. Delogu, Jose A. Rodríguez-Marroyo, Domingo M. Molina-Terrén

**Affiliations:** 1 VALFIS Research Group, Department of Physical Education and Sports Sciences, Institute of Biomedicine (IBIOMED), University of León, 24071 León, Spain; 2 Department of Science for Nature and Environmental Resources (DipNeT), University of Sassari, Sardinia, 07100 Sassari, Italy; 3 Department of Crops and Forest Sciences, School of Agrifood and Forestry Science and Engineering, University of Lleida, 25198 Lleida, Spain

**Keywords:** heat strain, heat stress, acclimatisation, mitigation strategies, wildland fire suppression, wildland firefighters, structural firefighters

## Abstract

This study aimed to assess current perceptions of heat stress, heat strain, acclimatisation and recovery practices in wildland fire suppression. A total of 1459 wildfire and structural firefighters, all involved in wildland fire suppression, completed an 18-question survey. Most participants (81.3%) reported heat strain as one of the main risks faced during wildland firefighting. Thermal strain is considered an important risk for health and safety in wildland firefighting. The best-valued heat strain mitigation strategies were those traditionally recommended in wildland fire suppression: (i) an adequate work/rest ratio (79.0%), (ii) acclimatisation (71.6%), (iii) enhancing body ventilation by opening protective clothing or removing helmets or gloves (63.5%), and (iv) drinking water and food supplementation (52.1%). Despite these results, only 22% of the participants reported carrying out acclimatisation in the workplace. The vast majority of the respondents (87.4%) consider active cooling strategies (i.e., ice slurry ingestion, ice vests, etc.) impractical in combating heat strain during wildfire suppression. We identified a gap between knowledge about heat strain, its mitigation strategies and the level of actual implementation of these practices in the workplace. Our results highlight the need to improve heat strain management and implement operational directives for acclimatisation and active cooling interventions.

## 1. Introduction

Wildland fire suppression presents a unique and diverse set of challenges for individuals from a physiological perspective [1,2]. Wildland firefighters (WFFs) perform demanding physical work (e.g., fireline construction, charged hose advance, hiking in sloped terrains) [3] for extended periods [1]. In addition, these activities are commonly undertaken in environmental conditions that combine high ambient temperatures, exposure to flame heat flux [4] and smoke inhalation [5]. The combination of all these factors increases worksite heat stress, facilitating increased body heat and increasing firefighters’ heat strain (i.e., higher heart rate, sweat rate and body temperature) [6]. Moreover, personal protective equipment (PPE) may exacerbate firefighters’ thermal strain, preventing heat transfer release and sweat evaporation [7]. The resulting restriction in body heat dissipation causes a rapid rise in body core temperature, which has been related to a decrease in physical performance, loss of cognitive performance [8] and increased risk of heat-related illness symptoms (i.e., headache, sudden muscle cramps, dizziness, nausea, vomiting and fainting) [9,10]. Furthermore, physiological and psychological fatigue can compromise work performance and increase the risk of injury [11] and workplace accidents such as fire entrapment [12,13].

Due to the implications of heat strain on the health and safety of firefighters, several studies have analysed different strategies aimed at mitigating heat strain [14,15,16,17,18,19]. The mitigation strategies recommended for structural and wildland firefighters rely on the expected heat stress level and the ease of putting them into practice in the workplace. On the one hand, structural firefighters have to extinguish fires in confined spaces in urban contexts where thermal environments can reach temperatures of up to 200 °C [20,21]. To ensure safety, structural firefighters wear highly encapsulating personal protective equipment (PPE) and wear self-contained breathing apparatus that limit work time to 20 min [22]. Wildland firefighters, on the other hand, perform long-lasting suppression efforts in open environments in remote areas [10], where ambient temperatures reach as high as ~80 °C [4]. With these differences in job assignments, forearm/wrist cooling [23,24], ice slurry ingestion [17,18] and immersion in cold water [14,17,19] have been suggested for heat strain recovery in structural firefighters. For WFFs, however, the most frequently suggested strategies to regulate body temperature are work and rest cycles, fluid intake guidelines and heat acclimatisation [2,15,16]. Considering that, in Europe and Latin America (LATAM), it is very common for structural firefighters to carry out wildland fire suppression, the question that remains unsolved is if structural fire brigades adapt their heat mitigation strategies, if any, considering the wildland fire suppression scenario, especially regarding acclimatisation protocols.

Despite firefighters’ health and safety consequences of heat strain, there is currently limited understanding of how these workers perceive it, its causal factors and the existing mitigation strategies. Recently, the perception of heat strain and recovery strategies have been analysed in Australian firefighters [25] This study reported that structural firefighting was the hottest operational activity experienced, followed by wildland firefighting. In addition, the study highlighted the relevance of cooling strategies to firefighters as a means of recovering from heat strain and fatigue. In line with these findings, a survey on occupational cooling practices conducted in 119 first responders’ departments in the United States showed that heat strain is considered the main risk to safety in occupational duties [26]. Likewise, the cooling recovery techniques were considered key strategies in optimising safety after deployments [26]. However, despite the importance given to heat mitigation strategies, it has been documented that a high percentage of first responders are not yet familiar with their use [27]. 

There seems to be a gap between scientific evidence and current end-user heat management practices, considering the observations from previous studies [28,29]. Understanding heat strain perception during wildland fire suppression can guide the development of operational and policy directives to enhance health and safety conditions. Furthermore, this information would help to optimise the design of recovery strategies to improve performance and reduce the risk of workplace heat strain. This is especially relevant in the current scenario, where climate change has been associated with a trend towards more extreme wildfire events in terms of geographical extent, duration, intensity and severity, thus posing firefighters with more dangerous working conditions [30,31]. To the best of our knowledge, no studies in this regard have been conducted in Southern Europe and Latin American areas despite the key importance of wildland fires in these areas. In these geographical contexts, differences in fire suppression practices, on-work culture and organisational management might lead to differences in heat strain perception and the implementation of its countermeasures. Therefore, this study aimed to gather current perceptions of heat stress, heat strain, acclimatisation and heat mitigation strategies in wildland fire suppression performed by wildland and structural firefighters across Southern Europe and Latin America.

## 2. Materials and Methods

### 2.1. Instrument

This was a cross-sectional study based on a self-reported survey. The questionnaire was jointly developed by specialists in heat stress and physiology from the University of León (Spain) and experts in wildland fires from the University of Lleida (Spain) and the University of Sassari (Italy) based on previous studies [25,26]. The first page of the questionnaire provided information on the study aims and characteristics and a consent item that was required to be filled out before data collection. The survey consisted of 18 questions grouped into two sections). The first section consisted of eight items focused on gathering demographical data such as age, gender, job position, years of experience in wildland fire suppression, country and the agency they work for (e.g., wildland or structural fire brigades). The second section aimed at gathering: (i) the significance given to heat strain as an occupational risk and to the heat stress sources, and (ii) perceptions and current implementation of acclimatisation protocols and heat strain mitigation methods in the field. In this second section, questions consisted of multiple-choice answers where participants had to appraise each proposed item independently using an 11-point Likert-type scale. In inquiries related to knowledge and personal beliefs, the Likert-type scale for each item ranged from 0 (i.e., strongly disagree/not influential at all) to 10 (i.e., strongly agree/decisive); in questions where participants were asked about their current on-work habits, the scale ranged from 0 (i.e., never) to 10 (i.e., every working day/on a regular basis). The survey instrument was pre-tested for length, clarity and comprehension with a convenience sample of 300 firefighters coming from different Spanish fire brigades (e.g., wildland and structural). In Italy, Portugal and LATAM, the first translated version of the survey was checked for clarity by asking the heads of the different organizations and fire departments to provide feedback. Minor modifications were made according to the feedback before the instrument was finalized.

### 2.2. Data Collation

All subjects participated voluntarily and their written consent was obtained before completing the questionnaire. The study was approved by the Ethics Committee of the University of León. Data were obtained through different procedures from WFFs and structural firefighters who were routinely involved in wildland fire suppression: (i) leveraging the on-the-job training sessions performed by internal wildland fire trainers in some regions of Spain (e.g., Región de Murcia, Castilla La Mancha and Catalonia). After the training session, the instructors performed a briefing about the purpose of this study. Then, the paper-based survey was distributed to participants; (ii) voluntary participation through a self-reported questionnaire in Google Forms. The survey was distributed through e-mail lists developed by the University of Lleida in the framework of the Master of Science in Wildland Fire Science and Integrative Management (MasterFUEGO, an academic program in the Spanish language); (iii) sending the questionnaire in Google Forms by personalised e-mail to different national and local fire agencies in Spain, Portugal, Italy, Argentina, Chile, Brazil and other countries, as well as professional associations such as the Forest Ranger Association and firefighter associations in the countries mentioned above. 

The questionnaire was distributed over different countries in Europe (Spain, Italy, Portugal) and Latin America (Argentina, Brazil, Chile, Mexico and others) and it was available in three different languages: Spanish, Italian and Portuguese (Brazilian and European Portuguese versions). All questionnaires were translated from Spanish into Italian and Portuguese by private translators and with the help of native specialists for technical items. Together with the questionnaire, the participants received instructions on filling it out. Support was offered for any questions or queries during the questionnaire. 

The data were collected from 31 January 2020–3 February 2021. Once the data were collected, the resulting database was then carefully reviewed to deal with any possible inconsistencies, duplicate information or dubious identifications. The subjects were contacted by e-mail for clarification when a discrepancy was found in the answers. When the level of inconsistency was high, and an explanatory response could not be found, the survey was removed from the database. In this sense, only 1% of the surveys gathered had to be removed from the database. The results obtained were analysed considering the country, the fire brigade type (i.e., wildland, structural firefighter) and level of responsibility (i.e., firefighter, middle management and senior management positions). Thus, within the wildland fire departments, subjects were classified as follows: wildland firefighter (WFF), middle management (WFMM) and forest range officer (FRO). On the other hand, the structural firefighters were divided into structural firefighters (SFF) and fire department middle management (FFMM). Finally, those subjects who held senior management positions, both in wildland fire and all-in-one fire agencies, were included in the group of high management (HM).

### 2.3. Data Analysis

Quantitative data are displayed as mean ± standard deviation. The prevalence of categorical data responses is reported in frequencies and percentages. The Kruskal–Wallis one-way analysis of variance was used to determine significant differences between group responses regarding quantitative data (i.e., country, type of fire brigade, and job position). If any differences were present, a post hoc pairwise test for multiple comparisons of mean rank sums was used to confirm significance (Bonferroni test). Pearson’s chi-square analysis was performed to assess whether the distributions of categorical variables differed from one another. A Fisher’s exact test was used when >20% of expected frequencies within each category were less than 5. Standardised residuals were assessed for significant differences between group responses and expected counts where significant associations were identified. The significance level was set at *p* < 0.05. Statistical analyses were performed using SPSS (v26, IBM Corporation, Armonk, NY, USA).

## 3. Results

### 3.1. Sample Characteristics

A total of 1459 responses were obtained, of which 81.9% were collected in Spain, 5.4% in Italy, and 3.8% in Portugal (Table 1). In Latin America, the answers were mainly obtained in Argentina (2.5%), Brazil (2.2%), Chile (1.9%) and Mexico (1.5%), with residual responses in other countries of Central and South America. The results gathered in America are shown in two groups: (i) Argentina (36) and (ii) the rest of the Latin American countries (LATAM) (99).

The participants were primarily male (91%) with a mean age of 41 ± 9 years. The most frequent age ranges were 41–50 years (34.7%) and 31–40 years (30.5%). Italian respondents were significantly (H = 73.808, *p* < 0.001) older (51 ± 11 years) compared to respondents from Spain, Argentina, LATAM (41 ± 9 years) and Portugal (38 ± 7 years). The job position showed significant differences (H = 133.013, *p* < 0.001) in the mean age. The WFF were the youngest (38 ± 8 yr), followed by the SFF (39 ± 8 years), WFMM (42 ± 10 years) and HM (43 ± 9 years), while the mean age of the FFMM (46 ± 6 years) and FRO (47 ± 7 years) was significantly older.

Firefighting experience averaged 15.4 ± 9.1 yr. The most frequent experience ranges were 11–15 years (22.1%) and 16–20 years (20.1%). The groups with the higher experience in wildland fire suppression (H = 149.689, *p* > 0.001) were FRO (21.5 ± 8.8 years), SFF (15.4 ± 7.4 years) and FFMM (18.3 ± 8.1 years), whereas the group with less experience was WFF (11.5 ± 8.3 years). The most frequently reported job positions were WFF and HM, while the least reported were SFF and FFMM (Table 1). A significant association between the country and the job held was found (χ^2^= 355.394, *p* < 0.001). The predominant responses were those of WFF in Spain, WFMM in Italy and Argentina, SFF in Portugal and HM in LATAM (Table 1).

### 3.2. Perception of Heat Strain

The heat strain was considered a relevant factor for firefighters’ health and safety during wildland fire suppression (7.7 ± 1.6). Most firefighters (86.8%) reported experiencing themselves or witnessing the effects of heat strain among their colleagues during wildland firefighting. The importance given to heat strain as a risk factor was significantly higher (H = 18.07; *p* < 0.001) in Argentina (9.6 ± 0.9) compared to Portugal (8.7 ± 1.7), Italy (8.6 ± 1.8) and Spain (8.5 ± 1.8) (Table 2). 

The majority of participants (χ^2^= 1110.9, *p* < 0.001) considered the thermal environment as hot (26.3%) or very hot (59.5%) with an average rating of 7.7 ± 1.9. The score given for the thermal environment was conditioned by the country (H = 41.65; *p* < 0.001) and the job position (H = 57.65; *p* < 0.001). Thus, the thermal environment was considered milder in Italy than in Spain, Portugal and LATAM. WFF and WFMM rated the thermal environment as hotter than HM, SFF, FFMM and FRO (Table 2).

The heat stress factors that participants considered most relevant (H = 65.26; *p* < 0.001) during wildland fire suppression were solar radiation, physical work, environmental temperature, the heat released from flames, and PPE (Table 3). The importance of these factors was conditioned by the firefighters’ country and job position. The environmental temperature was considered less relevant (H = 28.95; *p* < 0.001) in Portugal and Italy than in Spain and LATAM. In the same way, heat from flames was perceived as less important (H = 33.72; *p* < 0.001) in Portugal. HM and SFF perceived the sun’s radiation as the most important factor (H = 28.95; *p* < 0.001) compared to WFF and WFMM.

**Figure 1:**
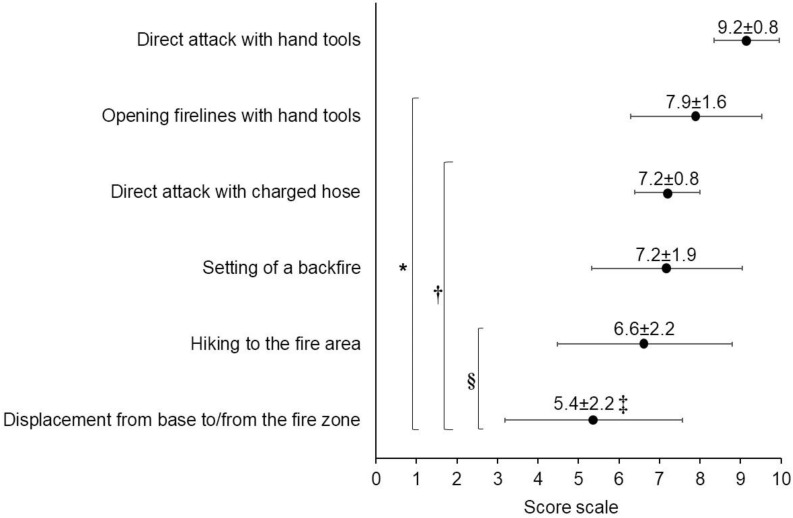
Overall perceived importance of tasks performed in wildland fire suppression in relation to heat stress (mean ± SD). Score: 0 = *not relevant at all* and 10 = *very important*. *: differences with direct attack with hand tools; †: differences with opening firelines with hand tools; §: differences with charged hose and backfire; ‡: differences between hiking to the fire area and displacement from base to/from the fire zone. All differences are significant at *p* < 0.001.

The tasks performed on deployments that firefighters believed had a more significant effect on heat strain than other tasks (H = 2651.81; *p* < 0.001) were direct attacks and opening firelines with hand tools (Figure 1). However, the degree of perceived significance varied by country. The direct attack with a charged hose (7.6 ± 1.4) was considered the most relevant task (H = 19.12; *p* < 0.001) in Portugal, while opening firelines with hand tools (7.3 ± 1.9) was considered less relevant (H = 14.53; *p* < 0.001) in this country compared to Italy (8.2 ± 1.7) and Spain (7.9 ± 1.6). Regarding the job position, SFF (7.6 ± 1.8) and FFMM (7.9 ± 1.4) selected the direct attack with a charged hose as the most important task (H = 29.05; *p* < 0.001). However, the direct attack with hand tools was more important (H = 13.05; *p* = 0.015) for WFF (9.2 ± 1.2) than SFF (8.7 ± 1.8).

Finally, respondents considered low physical fitness (9.0 ± 1.8), overweight (8.7 ± 1.8), hydration level, poor diet (8.6 ± 1.9) and alcohol consumption (8.2 ± 2.2) as factors that potentially make them more likely to suffer from heat-related illness during wildland firefighting. It was observed that physical fitness (H = 12.94; *p* < 0.05) and hydration level (H = 11.25; *p* < 0.05) were judged as more relevant for HM versus WFF (9.3 ± 1.6 vs. 8.9 ± 1.9 and 8.9 ± 1.6 vs. 8.2 ± 2.5, respectively). 

### 3.3. Heat Strain Mitigation Strategies

Seventy-one-point-six per cent of those surveyed considered heat acclimatisation an important strategy against heat strain, with an average score of 8.2 ± 1.8. In Italy, firefighters assigned a lower score (6.7 ± 2.2) to the need to be acclimatised to the heat (H = 46.52; *p* < 0.001) compared to the assessment given in the rest of the countries (~8 points). Acclimatisation was assessed significantly lower (H = 12.429, *p* = 0.029) in the SFF group (7.6 ± 2.2) compared to the WFF (8.2 ± 1.8), WFMM (8.1 ± 1.8) and HM (8.3 ± 2.0).

**Figure 2:**
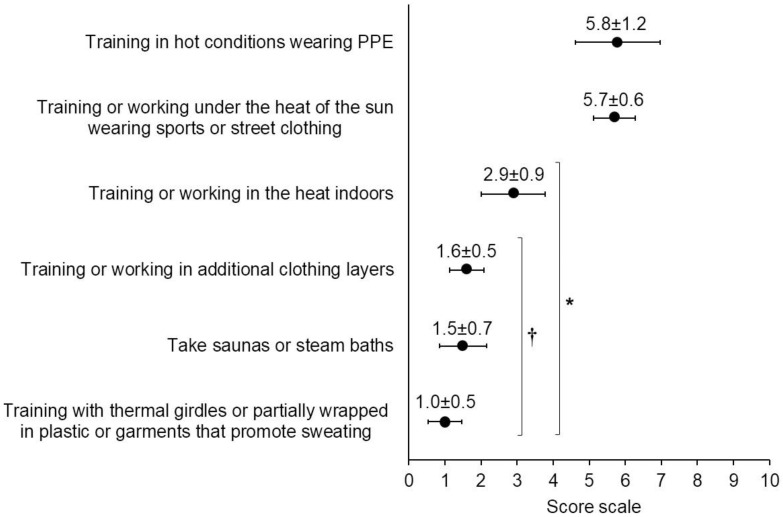
Overall scores given to the acclimatisation strategies (mean ± SD). Score: 0 = *never*, 10 = *on a regular basis*. *: differences between training in hot conditions wearing PPE and training under the heat of the sun wearing sports or street clothing; †: differences with training or working indoors with heat. All differences are significant at *p* < 0.001.

Consistent with the above findings, the statement “which of the following strategies do you take to acclimatise to the heat and excessive sweating” obtained an average score of 3.8 ± 3.6. A third of the participants (33.0%) rated this item with 0 points (e.g., never), and only 22% gave this aspect 10 points (e.g., on a regular basis). The overall scores of the implementation of acclimatisation strategies are shown in Figure 2. The best-valued acclimatisation strategies (H = 2168.37; *p* < 0.001) were training in the heat wearing PPE, and training in sportswear in hot conditions. Different perceptions according to the country (H = 26.006, *p* < 0.001) and job position (H = 87.457, *p* < 0.001) were found. In this regard, the implementation of acclimatisation protocols was perceived as less important in Italy (1.9 ± 2.6) than in Spain (4.0 ± 3.9), Argentina (3.9 ± 3.9) and LATAM (4.0 ± 3.9). It was also less relevant for FRO (1.5 ± 2.5) and SFF (2.4 ± 3.0) than for WFF (4.4 ± 3.7), WFMM (4.2 ± 3.5) and HM (4.0 ± 3.3). 

**Figure 3:**
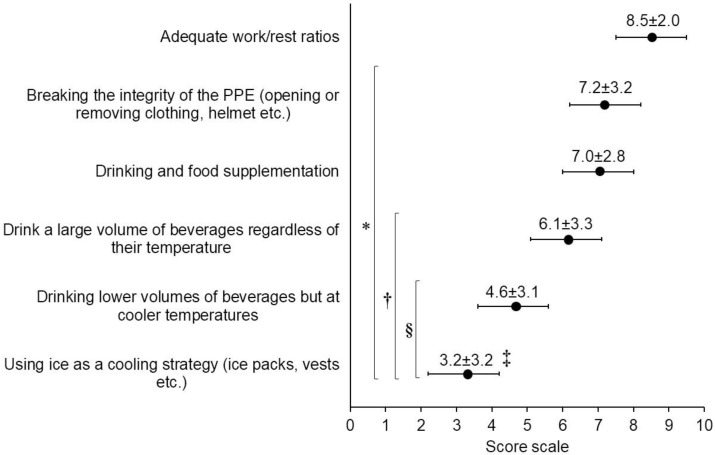
Perceived effectiveness of the heat strain mitigation strategies during wildland fire suppression (mean ± SD). Score: 0 = *not effective at all* and 10 = *totally effective*. *: differences with adequate work/rest ratios; †: differences with breaking integrity of PPE and drinking and food supplementation; §: differences with drinking a large volume of beverages regardless of their temperature; ‡: differences with drinking lower volumes of beverages but at cooler temperatures. All differences are significant at *p* < 0.001.

The perceived effectiveness of other heat strain mitigation strategies is provided in Figure 3. An adequate work-to-rest ratio was assessed as the most effective (H = 2209.0; *p* < 0.001), followed by opening protective clothing and/or removing helmets, and water intake with food supplementation. The volume of ingested fluid and the temperature of the consumed beverages were perceived as less important heat strain countermeasures. Active cooling did not reach the general approval, so this strategy was perceived as the less effective one, with the score assigned to this strategy being 3.2 ± 3.2. The significance given to ice-cooling strategies by SFF (4.1 ± 3.3) was higher (H = 27.893) than that of WFMM (2.8 ± 3.1) and HM (2.6 ± 2.9). FRO judged fluid intake and food supplementation as a less (H = 20.563, *p* < 0.001) effective strategy (6.0 ± 3.2) versus WFF (7.0 ± 2.9), WFMM (7.0 ± 2.7), SFF (7.6 ± 2.4) and FFMM (7.9 ± 1.8).

## 4. Discussion

This study is the first to gather perceptions of heat strain, heat stress, acclimatisation and mitigation practices in wildland fire suppression across Southern Europe and Latin America. Most respondents (~90%) considered heat strain a significant risk to their health and safety while performing wildland fire suppression activities. Heat stress factors, such as the sun’s radiation, and suppression tasks near flames are considered the most stressful factors. Although acclimatisation is regarded as an effective strategy against heat strain, its application in the field seems limited. In contrast, heat strain mitigation practices such as work/rest ratios or hydration protocols are preferred in wildland fire suppression. Active cooling methods such as ice slurry ingestion, ice vests and ice packs to recover from heat strain were perceived as less effective.

Overall, the importance of heat strain as an occupational hazard found in our study is similar to those previously reported in the USA [26] and Australia [25]. A survey distributed among 180 first responders’ departments in the US showed that 99% of participants considered heat illness a relevant risk to their safety [26]. Similarly, Fullagar et al. (2021) have recently suggested that heat strain is a significant risk factor during firefighting tasks. These findings collectively add more evidence to current knowledge on the relevance of heat strain in workplaces [29,32]. 

Firefighters identified environmental conditions (e.g., solar radiation, ambient temperature, flames), physical work and PPE as the primary sources of heat stress. This is something to be expected since the interrelation of these factors defines the net heat load to which firefighters are exposed (e.g., heat stress) [33]. Several studies have shown how firefighters’ thermophysiological response is affected by the nature of the tasks performed [1,34,35] and how environmental conditions [2,4] and PPE [7,22,36,37] can reduce the body heat release, contributing toward increasing the physiological strain further. Therefore, it seems logical that the firefighters of this study perceived direct attack (i.e., closer to the flames) as the task that most influences heat strain. Indeed, the significant impact of external heat sources on firefighters’ heat strain perception has recently been reported [25]. It is worth noting the less consideration given to heat stress factors in Italy and Portugal. For Italian respondents, the results may be linked to the fact that most of the participants were WFMM. This job position mainly involves carrying out supervision of the team; therefore, physical work and exposure to environmental factors are not as direct as for WFF. On the other hand, the scores obtained in Portugal are probably because wildland fire suppression is performed by structural fire brigades mainly using hose laying, which means working at a certain distance from the front of the flames. In the rest of the countries, direct attack with hand tools is the suppression activity most often performed.

Contrary to what may be expected, structural (i.e., SFF and FFMM) vs. wildland (i.e., WFF and WFMM) firefighters considered the role of PPE on heat strain less relevant. The mass of PPE typically worn by structural firefighters (~20 kg) is greater than that of WFFs (~16 kg) [7]. In addition, the thermal insulation of structural firefighters’ protective clothing (~0.50 m2·K·W^−^^1^) [38] is on average twofold greater than that found in WFFs’ (~0.23 m2·K·W^−^^1^) [39]. Structural firefighters’ PPE could lead to higher metabolic heat production (by restricting movement efficiency and increasing weight) [40] and lower heat loss efficiency (by a higher encapsulation) [22], which would increase the subjects’ thermal strain. Therefore, it would have been expected that PPE had a greater influence on structural firefighters’ perception of heat strain [26,41,42]. A possible explanation for the obtained result could be the habituation of structural firefighters to use PPE in more extreme conditions. It has been reported that these subjects may withstand ambient temperatures between 50–100 °C, reaching maximum exposures above 200 °C [20,21]. However, in wildland fires, the environmental temperatures are ostensibly lower (i.e., ~32 and ~80 °C of mean and maximum environmental temperature, respectively) [4], which could have conditioned the subjective perception that these subjects gave to the effect that PPE has on heat strain during wildland firefighting. 

Respondents recognised heat acclimatisation as a critical strategy for individual and team safety with ~72% considering heat acclimatisation an effective strategy to mitigate heat strain. This outcome might be linked to the fact that in scientific literature, acclimatisation has long been acknowledged as an effective mitigation strategy for firefighters [2,16,43,44]. Based on these findings, acclimatisation has been recommended by government agencies as one of the heat strain mitigation strategies to apply in occupational settings [33] and in wildland fire fighting, specifically [45]. However, our study showed that heat acclimatisation protocols are not widespread among the analysed countries and fire brigades, highlighting the mismatch between theory and on-field practice [25,26]. Acclimatisation seems to be more accepted and implemented among wildland than structural fire brigades. In this regard, the best-perceived acclimatisation strategies coincided with those recommended for WFFs [45] were training in hot conditions while wearing PPE or sports clothing. 

In line with previous findings, the vast majority of the participants (~80%) stated that the heat strain alleviation practices most frequently followed are an appropriate work/rest ratio, removing PPE items (e.g., helmet, flash hood and jacket), and drinking water with food supplementation, aiming at electrolyte and carbohydrate reposition. These strategies were possibly selected due to their ease of implementation during wildland firefighting [25]. This observation might be linked, firstly, to the fact that these mitigation strategies have been widely advised in wildland fire suppression [45] and, secondly, because of the relevance that wildland fire brigades had in the sample, since up to 61% of respondents were WFF and WFMM. Notwithstanding this, our results agreed with those of [25] obtained with structural firefighters in Australia. The authors reported that the top three recovery strategies performed by structural firefighters during their deployments were: sitting in the shade (93%), ingesting cold water (90%) and removing the helmet, flash hood and jacket (89%). In our study, active cooling strategies (e.g., ice vests or gel packs) were perceived as less applicable by the respondents (Figure 3), even though these methods have shown to be effective in heat strain alleviation before, during and after firefighters’ deployments [17,18]. Various factors such as availability, economic cost, logistics, and knowledge could explain these views [26]. 

The present study has potential limitations. First, a survey reflects the current practice at a particular time. The dynamic nature of the assimilation of new information or findings and public, political or supervisory pressures could condition the results in the near future. Second, there is the possibility of participant self-selection bias and cross-over regarding firefighter roles and ranks, which could affect their perceptions. Third, participants were asked to provide their retrospectives of working and training practices, which may have been influenced by different factors such as recent experiences and workplace culture. Fourth, respondents were, in their majority, WFF and HM coming from Spain, which might have influenced the results. Therefore, although the results of this study can help to provide insight into firefighters’ views, they should be limited to the agencies or departments included.

## 5. Conclusions

This research that surveys 1459 responders is an approach to firefighters’ views about heat strain, heat stress, heat acclimatisation and heat strain mitigation while suppressing wildland fires across Southern Europe and Latin America. The thermal strain was considered a relevant risk for health and safety during wildland firefighting. The heat from flames, direct attacks with hand tools, and protective clothing were the primary sources of heat stress. Even though heat strain is considered a relevant risk by consensus, there is a marked asymmetry in heat strain management in the field. Heat acclimatisation was appraised as an effective strategy to cope with thermal strain, especially by wildland, rather than structural, firefighters. Despite this, our results highlight that the implementation of acclimatisation practices in the fire services was lacking. The best-considered heat strain mitigation strategies during fire deployments were the most readily and easily applicable, such as work/rest ratios and hydration protocols. The results show the need to update existing workplace heat stress and heat strain management, especially regarding acclimatisation and heat strain mitigation practices (e.g., work/rest ratios and active cooling interventions).

## Figures and Tables

**Table 1:** Responses obtained by job position and country. Values expressed as absolute count (percentages).

	WFF	WFMM	FRO	SFF	FFMM	HM	Total
Spain	561 (47.2) *	199 (16.7)	113 (9.5)	61 (5.1)	47 (4.0)	208 (17.5)	1189 (81.5)
Italy	25 (31.6)	28 (35.4) *	0	5 (6.3)	1 (1.3)	20 (25.3)	79 (5.4)
Portugal	0	7 (12.5)	0	30 (53.6) *	7 (12.5)	12 (21.4)	56 (3.8)
Argentina	9 (25.0)	16 (28.6) *	4 (7.1)	2 (3.6)	1 (1.8)	4 (7.1)	36 (2.5)
LATAM	20 (20.2)	25 (25.3)	0	2 (2.0)	1 (1.0)	51 (51.5) *	99 (6.8)
**Total**	**615 (42.2)**	**275 (18.8)**	**117 (8.0)**	**100 (6.9)**	**57 (3.9)**	**295 (20.2)**	**1459 (100)**

WFF: wildland firefighter; WFMM: wildland fire department middle management; FRO: forest range officer; SFF: structural firefighter; FFMM: structural fire department middle management; HM: high management (combining wildland and structural fire departments). *: higher count in this category than the expected count (*p* < 0.05).

**Table 2:** Importance given as risks factors to heat strain and the thermal environment during wildland fire suppression by country and job position. Values expressed as mean ± SD.

	Heat Strain	Thermal Environment
Spain	8.5 ± 1.8	7.7 ± 1.6
Italy	8.6 ± 1.8	6.6 ± 1.5 ^‡,§,^*
Portugal	8.7 ± 1.7	7.7 ± 1.5
Argentina	9.6 ± 0.6 ^‡,†,§^	7.4 ± 2.2
LATAM	9.0 ± 1.6 ^‡,†^	8.0 ± 2.0
WFF	8.6 ± 1.9	7.9 ± 1.6 ^1,2,3,4^
SFF	8.8 ± 1.9	7.4 ± 1.6
WFMM	8.5 ± 1.7	7.8 ± 1.7 ^1,2,3,4^
FRO	8.6 ± 1.10	6.9 ± 1.6
FFMM	9.1 ± 1.4	7.2 ± 1.5
HM	8.8 ± 1.7	7.6 ± 1.7
**Total**	**8.7 ± 1.8**	**7.7 ± 1.7**

LATAM: Latin America except for Argentina; WFF: wildland firefighter; WFMM: wildland fire department middle management; FRO: range forest officer; SFF: structural firefighter; FFMM: structural fire department middle management; HM: high management (combining wildland and structural fire departments). Score: 0 = *not relevant at all* and 10 = *very important*. ^‡^: differences with Spain; ^†^: differences with Italy; ^§^: differences with Portugal; *: differences with LATAM. ^1^: Differences with HM; ^2^: differences with FRO; ^3^: differences with FF; ^4^: differences with FFMM. Differences are significant at *p* < 0.001.

**Table 3:** Scores assigned to the different factors related to thermal stress during wildland fire suppression by country and job position (n = 1459). Values expressed as mean ± SD.

	Ambient Temperature	Flames	Sun Radiation	Wind	Physical Exertion	PPE	Smoke
Spain	8.1 ± 1.6 ^C,D,E,G^	8.0 ± 1.6 *^,C,D,E,G^	9.1 ± 1.2 ^D,E,F,G^	6.4 ± 2.2 ^E,F,G^	8.5 ± 1.4 ^F,G^	7.9 ± 1.6 ^G^	7.4 ± 2.3
Italy	7.6 ± 1.6 *^,C,D,E^	7.6 ± 1.6 *^,C,D,E^	8.8 ± 1.4 ^D,F,G^	5.8 ± 2.3 *^,E,F,G^	8.7 ± 1.2 ^F,G^	7.8 ± 1.7 ^G^	7.0 ± 2.7
Portugal	7.4 ± 1.8 *^,‡,C^	7.2 ± 2.2 *^,‡,§,C^	8.8 ± 1.7 ^D^	6.9 ± 2.3 ^†^	8.0 ± 2.0	7.6 ± 2.1	7.8 ± 2.1
Argentina	7.9 ± 1.7 ^D^	8.3 ± 1.5 *^,D^	9.1 ± 1.7 ^D,F,G^	6.2 ± 2.6	8.4 ± 1.6	7.3 ± 2.0 ^C^	7.2 ± 2.4 ^C^
LATAM	8.5 ± 1.6 ^G^	8.7 ± 1.4 ^F,G^	9.2 ± 1.3 ^D,E,F,G^	7.1 ± 2.2 ^E,F^	8.3 ± 1.6	7.9 ± 1.9	7.7 ± 2.3
WFF	8.1 ± 1.6 ^C,D,E,G^	8.1 ± 1.6 ^C,G^	9.2 ± 1.3 ^1,3,D,E,F,G^	6.5 ± 2.2 ^E,F,G^	8.4 ± 1.4 ^F,G^	7.8 ± 1.7 ^C,D,E^	7.8 ± 2.2 ^1,2^
SFF	8.0 ± 1.8 ^C,D^	7.9 ± 1.8 ^C,D^	8.8 ± 1.6 ^D,F,G^	6.8 ± 2.1 ^E,F,G^	8.4 ± 1.7 ^G^	7.9 ± 1.8	7.7 ± 2.2 ^1^
WFMM	8.0 ± 1.6 ^C,D,F,G^	7.8 ± 1.6 ^C,D,E^	9.2 ± 1.2 ^1,3,D,E,F,G^	6.4 ± 2.3 ^A,B,C^	8.5 ± 1.4 ^F,G^	7.9 ± 1.7	7.4 ± 2.41
FRO	8.1 ± 1.4 ^C,D,E,G^	8.0 ± 1.6 ^C,D,E,G^	9.1 ± 1.1 ^F,G^	6.0 ± 2.2 ^E,F,G^	8.7 ± 1.2 ^F,G^	7.7 ± 1.5	7.1 ± 2.11
FFMM	8.1 ± 1.6 ^C,D^	8.1 ± 1.4 ^C,D^	9.1 ± 1.0 ^D,G^	7.1 ± 1.72 ^E,F^	8.5 ± 1.5 ^G^	8.2 ± 1.6 ^D^	7.2 ± 2.02 ^A,C,E,F^
HM	8.0 ± 1.7 ^C,D,E,G^	8.1 ± 1.5 ^C,D,G^	8.9 ± 1.4 ^D,G^	6.3 ± 2.3 ^E,F^	8.6 ± 1.5 ^F,G^	7.8 ± 1.7 ^G^	6.7 ± 2.5 ^A,B,C,E,F^
**Total**	**8.0 ± 1.7 ^C,D,E,F,G^**	**8.0 ± 1.4 ^C,D,E,F,G^**	**9.0 ± 1.6 ^D,E,F,G^**	**6.5 ± 2.2 ^E,F,G^**	**8.5 ± 1.5 ^F,G^**	**7.8 ± 1.8 ^G^**	**7.3 ± 2.3 ^A,B,C,E,F^**

LATAM: Latin America except for Argentina; WFF: wildland firefighter; WFMM: wildland fire department middle management; FRO: range forest officer; SFF: structural firefighter; FFMM: structural fire department middle management; HM: high management (combining wildland and structural fire departments). PPE: personal protective equipment. *: differences with LATAM; ^†^: differences with Italy; ^‡^: differences with Spain; ^§^: differences with Argentina. ^1^: Differences with HM; ^2^: differences with FRO; ^3^ differences with SFF. Score: 0 = *not relevant at all* and 10 = *very important.* Differences between rows are significant at *p* < 0.001. ^A^: difference with ambient temperature; ^B^: difference with flames; ^C^: differences with heat from the sun; ^D^: differences with wind; ^E^: differences with physical work; ^F^: differences with PPE; ^G:^ differences with smoke. Differences between columns are significant at *p* < 0.05.

## Data Availability

The data presented in this study are available on request from the corresponding author. The data are not publicly available due to privacy restrictions.
